# Editorial: Community series in plants and microbial communities: diversity, pathogens and biological control, volume II

**DOI:** 10.3389/fmicb.2023.1199586

**Published:** 2023-12-01

**Authors:** Yong Wang, Md. Amin Uddin Mridha

**Affiliations:** ^1^Department of Plant Pathology, Agriculture College, Guizhou University, Huaxi, China; ^2^Faculty of Graduate Studies, Daffodil International University, Daffodil Smart City, Savar, Dhaka, Bangladesh

**Keywords:** bacteria, biological control, diversity, fungi, plant pathogens

We are grateful for the opportunity to act as the guest editors of this Research Topic, referring to contributions to microbial diversity, plant pathogens, and biocontrol. It was very encouraging to receive a large number of high-quality manuscripts covering microbial diversity, including fungi, bacteria, and viruses. Most of the manuscripts dealt with fungal pathogens causing different diseases in diversified plants. A few articles dealt with bacteria and viruses.

*Magnaporthe oryzae* is the most important fungal pathogen of rice (Donofrio et al., 2014). In this Research Topic, one article mentioned that mitochondrial carbonic anhydrase had the function for its conidiogenesis and pathogenesis (Dang et al.). Zhang P. et al. proposed that *M. oryzae* and *C. graminicola* belonged to the hemibiotrophic pathogens, and their sporulation and necrotrophic pathogenesis were linked with aspartate transaminase. Moreover, the crude lipopeptide of one *Streptomyces* strain had biocontrol potential against *M. oryzae*, and complete genome analysis indicated a number of key functional gene clusters that contribute to the biosynthesis of active secondary metabolites (Liu et al.). Additionally, it was reported that the high osmolarity glycerol of *Aspergillus cristatus* (Achog1) was present in the reaction during the processes of asexual sporulation, stress responses, and pigmentation (Shao et al.).

One article reported the biological control of maize *Fusarium* stalk rot by *Bacillus siamensis* isolated from rhizosphere soil under field conditions (Zhang K. et al.). Another reported on the taxonomy and control of *Trichoderma hymenopellicola*, which was reported as a novel taxon based on morphological comparison and phylogenetic analyses and can cause green mold disease on *Hymenopellis raphanipes*. A prochloraz-manganese chloride complex and Propiconazole were very effective in inhibiting the pathogen (Zeng et al.). Some authors mentioned the biological characteristics and fungicide sensitivity of *Colletotrichum godetiae*, which caused fruit rot in sweet cherry in the Southwest of China (Peng et al.). In Serbia, there was a high distribution of *Alternaria solani* and *A. protenta*, which revealed a new insight into the occurrence of early blight disease in potatoes (Ivanović et al.).

Phyllosphere microorganisms and potential pathogens of tobacco leaves were detected, which will be important in controlling tobacco diseases (Xiang et al.). Dwarf bunt is an important disease in wheat (Xu et al., 2021). Rhizosphere microbial communities were featured for disease incidence, and the concentration of difenoconazole fungicide was optimized as a control measure, helpful in the management of this disease (Jia et al.). Dong et al. introduced the microbial communities of rhizosphere soils and barks of *Eucommia ulmoides*, referring to structure, core microbiota, and function.

Innovative and interesting research findings were published in this Research Topic that recommend the best combination of NPK fertilizer, which increased the output and quality of panax ginseng and also influenced the diversity and structure of rhizosphere fungal communities (Sun J. et al.). Another research finding introduced an interactive mechanism to powerfully regulate the impact of plants on soil bacterial diversity (Wang Y. et al.). Under different habitats, the endophytic fungal community of *Dendrobium nobile* can influence the biosynthesis of dendrobine, which was also a very good piece of information (Li L. et al.).

Four articles referring to bacteria were collected in this Research Topic. By analyzing endophytic bacteria, Sun N. et al. introduced microbial diversity and the ecological actions and interaction mechanisms between white radish and endophyte resources. Under low osmostress, *Xanthomonas citri* was able to activate a novel β-glucosidase to reverse fitness deficiency (Li K. et al.), and one *Bacillus* strain obtained from tomato rhizosphere soil showed potential against *Meloidogyne incognita* (Du et al.). *Bacillus velezensis* isolated from lily can be used as a potential biofertilizer and biocontrol agent, which was functionally characterized by whole genome sequencing (Li B. et al.).

Subjects about viruses were also mentioned in this Research Topic. A novel partitivirus in the Betapartitivirus genus isolated from *Rhizoctonia solani* was established by phylogenetic analyses, including two dsRNA fragments encoding an RNA-dependent RNA polymerase and a coat protein (Chen et al.). Reyes et al. revealed new viral species by characterizing causal agents of cocoa pod rot disease and the associated microbiota dynamics of the pathogen *Moniliophthora roreri*. Physiological and transcriptomic analyses indicated that gene networks of tomato plants treated by SA and JA can heighten resistance against tomato yellow leaf curl virus (TYLCV) during the infecting process (Wang P. et al.). Maize chlorotic mottle virus can produce a coat protein related to tobacco mosaic virus hybrids in terms of symptom development and systemic movement (Zhang C. et al.).

Two other topics were covered in this Research Topic, for example, *Ricinus communis* seedlings, as a model plant for phloem, were utilized to clarify the uptake and transport mechanisms of the antibiotic fungicide Kasugamycin, which was a very informative article (Zhang H. et al.). The U-box E3 ubiquitin ligase gene family in *Sorghum bicolor* was identified and characterized by genome-wide analyses, and many sorghum U-box genes might be involved in many stress responses, as determined by multiplex approaches, viz., comprehensive analysis of promoters, expression profiling, and gene co-regulation networks (Fang et al.).

We would like to convey our thanks and gratitude to the authors who have published their valuable research findings in Frontiers in Microbiology.

## Author contributions

MM finished the first draft. YW gave some comments. All authors contributed to the article and approved the submitted version.
